# A real-world data analysis of ocular adverse events linked to anti-VEGF drugs: a WHO-VigiAccess study

**DOI:** 10.3389/fphar.2024.1398783

**Published:** 2024-07-30

**Authors:** Chen Li, Yicheng Lu, Ziyue Song, Yueqi Liu

**Affiliations:** Department of Ophthalmology, The First Affiliated Hospital of Soochow University, Suzhou, Jiangsu, China

**Keywords:** vascular endothelial growth factor, vascular endothelial growth factor inhibitor, adverse drug reaction, pharmacovigilance, spontaneous reporting, VigiAccess

## Abstract

**Introduction:**

Vascular endothelial growth factor (VEGF) is key to wet age-related macular degeneration (wAMD). Anti-VEGF drugs are the main treatment in clinics. This study assessed ocular adverse events (AE) from anti-VEGF drugs in VigiAccess, WHO’s database, and compared adverse drug reaction (ADR) profiles of four drugs to aid personalized treatment choices for optimal benefit and safety.

**Methods:**

The design was a descriptive retrospective study. We observed four anti-VEGF drugs commonly used in the clinical treatment of wAMD, and their ADR reports came from WHO-VigiAccess. The collected data included the age group, gender, and regional data, as well as the data of disease systems and symptoms caused by ADR recorded in the annual ADR reports and reports received by the WHO. We observed the overall characteristics of the ADR reports of these drugs, then explored the distribution of 27 SOCs of these drugs. Subsequently, we compared the most common ocular ADRs of the drugs. Finally, we compared the commonalities and differences of ocular ADRs related to the drugs.

**Results:**

Overall, 57,779 AE associated with the four anti-VEGF drugs were reported. The results showed that the number of females experiencing ADRs (67.83%) was significantly higher than males (32.17%), the age group with the highest reported incidence was over 75 years old. More than half of the ADR reports came from the Americas (50.86%). The five most common types of AE were: eye disorders (43.56%), general disorders and administration site conditions (34.47%), injury poisoning and procedural complications (13.36%), infections and infestations (11.61%), nervous system disorders (9.99%). Compared with the other three inhibitors, brolucizumab had a significantly higher rate of ocular ADR reports. The most common ocular ADRs of these four anti-VEGF drugs were mostly related to visual impairment, vision blurred, and blindness. However, there is still a disparity of ADRs between different drugs.

**Conclusion:**

The presence of ocular AEs when using anti-VEGF drugs to treat wAMD in clinical practice should attract clinical attention. Clinicians should use these expensive drugs more rationally based on the characteristics of ADRs and develop personalized treatment plans for patients.

## Introduction

Age-related macular degeneration (AMD) is a common irreversible blinding eye disease in the elderly, with the onset age mostly above 45 years and the incidence increasing with age. Early symptoms include decreased central vision and distorted vision, which, if not treated in time, can lead to severe impairment of central vision or even blindness (Guymer and Campbell, 2023). Currently, with the intensification of global population aging, it is estimated that by 2040, the global number of AMD patients will reach 288 million (Wong et al., 2014). Based on pathological changes, AMD can be divided into wet AMD (wAMD) and dry AMD. wAMD, also known as neovascular AMD, is characterized by choroidal neovascularization (CNV) or retinal neovascularization, accompanied by retinal bleeding, edema, and scarring. wAMD accounts for about 10%–15% of AMD patients, but its harm to vision is far greater than that of dry AMD (Shome et al., 2023). In the past, there were no effective drugs for wAMD, and it could only be treated by photodynamic therapy or vitrectomy, but the effects of these methods were not significant. Not until 2006 did the emergence of anti-VEGF biotherapy break the state of no cure. Today, anti-VEGF biotherapy has become the first-line treatment for wAMD (ElSheikh et al., 2022; Fleckenstein et al., 2024).

The vascular endothelial growth factor (VEGF) is an important angiogenic regulator that acts on vascular endothelial cells. It has multiple functions, including promoting the proliferation and differentiation of endothelial cells, inducing angiogenesis, and increasing the permeability of microvessels. VEGF plays a crucial role in maintaining the integrity of the vascular wall. However, the overexpression of VEGF can lead to abnormal vascular proliferation, which often results in wAMD and other fundus diseases within the eye. The anti-VEGF currently approved by the FDA for wAMD and other fundus diseases mainly include ranibizumab, aflibercept, brolucizumab, and faricimab. Ranibizumab was approved by the FDA in 2006 for the treatment of wAMD. Its main mechanism is to target and inhibit the VEGF-A receptor, binding to the VEGF-A subtype with high affinity, thereby inhibiting the binding of VEGF-A to its receptors VEGFR-1 and VEGFR-2. As it is a single-chain antibody with only the Fab segment, it has a shorter half-life and is less likely to activate the immune system, making it an ideal choice for the treatment of fundus diseases (Strzalka-Mrozik et al., 2024). Aflibercept is a soluble decoy receptor that can bind to VEGF-A, VEGF-B and placental growth factor (PLGF), inhibiting the binding and activation of endogenous VEGF receptors with VEGF-A and PLGF. Aflibercept was FDA-approved as a treatment for wAMD in 2011 (Heier et al., 2012). Brolucizumab is a human single-chain antibody fragment. It has inhibitory effects and high affinity for all VEGF-A subtypes, blocking the VEGF pathway by inhibiting ligand-receptor interactions, thereby inhibiting the formation of neovascular lesions, endothelial cell proliferation, and vascular permeability. Because it retains only two variable regions, it is smaller in size than ranibizumab, has higher affinity, faster tissue penetration, and is suitable for ocular administration. Brolucizumab was FDA-approved as a treatment for wAMD in 2019 (Pearce et al., 2022). Faricimab is a novel bispecific antibody that can simultaneously inhibit VEGF and angiopoietin-2 (ANG-2), two important angiogenesis signaling pathways, thereby more effectively regulating angiogenesis and vascular permeability. Faricimab was first FDA-approved in January 2022 for the treatment of wAMD (Stanga et al., 2023).

Anti-VEGF biotherapy for wAMD often requires regular and repeated intravitreal injections. Although the drug clinical trials have been very strict before marketing, due to different environments, it is impossible to identify the drug safety in the real world from drug clinical trials (Gagliardi et al., 2022). Therefore, the evaluation of drug safety based on real-world large sample data is worth further study. Currently, obtaining real-world drug safety data through spontaneous reporting systems (SRS) is a more authoritative and reliable method. The Uppsala Monitoring Center (UMC) on behalf of the World Health Organization (WHO) ’s Programme for International Drug Monitoring (PIDM) has collected adverse drug reaction (ADR) data worldwide. Up to 2018, UMC has collected and stored more than 20 million ADR reports from over 170 countries in the global spontaneous reporting project VigiBase. VigiAccess is a user-friendly web application that allows the public to freely access VigiBase and obtain ADR information related to drugs from around the world (Watson et al., 2018; Habarugira and Figueras, 2021). This study retrieved four anti-VEGF drugs approved by the US Food and Drug Administration (FDA) for the treatment of wAMD, including ranibizumab, aflibercept, brolucizumab, and faricimab. To compare the differences in the occurrence of related ocular ADRs of these four drugs, we performed a descriptive study of spontaneously reported ADRs in the VigiAccess and compared the reporting rates of ocular ADRs between the drugs. It is worth mentioning that there has been little research on the ADRs of these anti-VEGF drugs in the real world, especially the studies on their ocular ADRs are even fewer, where they are mainly used in patients with eye diseases. The results of this study will provide a theoretical basis for clinical doctors to compare different drugs and provide personalized treatment plans for patients.

## Materials and methods

Table 1 presents the basic information of four anti-VEGF drugs used for the treatment of wAMD. Structurally, each of the four drugs has unique characteristics. For instance, ranibizumab is a Fab antibody fragment, aflibercept is a fusion protein, brolucizumab is a single chain antibody fragment variable (scFv), and faricimab is a bispecific antibody. The mechanism of action also varies among these drugs. Ranibizumab and brolucizumab primarily bind with VEGF-A, while aflibercept can block VEGF-A, VEGF-B, and PLGF simultaneously, and faricimab can inhibit both VEGF-A and ANG-2. In terms of ophthalmic therapeutic indications, LUCENTIS^®^ (ranibizumab) is mainly used for the treatment of wAMD, diabetic macular edema (DME), retinal vein occlusion (RVO), secondary macular edema (ME), CNV, diabetic retinopathy (DR), and retinopathy of prematurity (ROP). EYLEA^®^ (aflibercept) can be used for wAMD, DME, RVO, DR, and ROP. Both BEOVU^®^ (brolucizumab) and VABYSMO^®^ (faricimab) can be primarily used for wAMD and DME.

**TABLE 1:** The basic information of four anti-VEGF drugs.

Drug name	Structure	Drug targets	Main eye conditions	First marketing time
Ranibizumab- LUCENTIS^®^	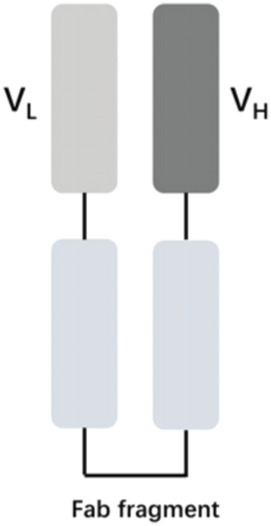	VEGF-A	wAMD, DME, RVO, secondary ME, CNV, DR, and ROP	2006
Aflibercept- EYLEA^®^	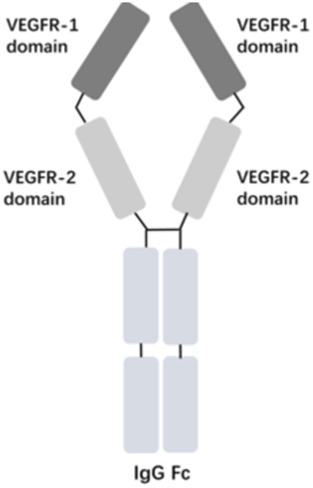	VEGF-A, VEGF-B and PLGF	wAMD, DME, RVO, DR, and ROP	2011
Brolucizumab- BEOVU^®^	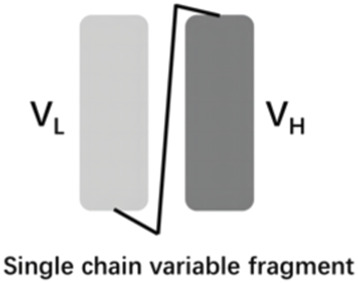	VEGF-A	wAMD and DME	2019
Faricimab-VABYSMO^®^	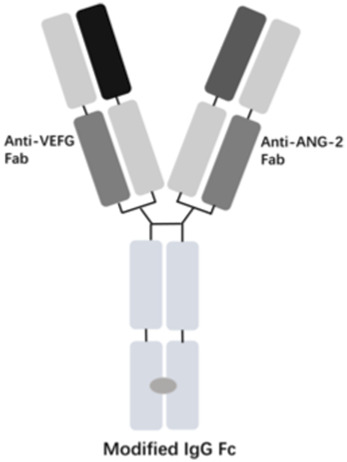	VEGF-A and ANG-2	wAMD and DME	2022

VEGF, vascular endothelial growth factor; wAMD, wet age-related macular degeneration; DME, diabetic macular edema; RVO, retinal vein occlusion; ME, macular edema; CNV, choroidal neovascularization; DR, diabetic retinopathy; ROP, retinopathy of prematurity; PLGF, placental growth factor; ANG-2, angiopoietin-2.

### Data source

All data were obtained from the WHO-VigiAccess website (https://www.vigiaccess.org). We searched for adverse events (AEs) following the use of each drug from 1 January, 2000 to 28 January, 2024, according to the generic and brand names of the drugs. The WHO’s information system collected data on age groups, gender, reporting year, and major continents worldwide. Descriptive data were calculated using Excel 2021.

WHO-VigiAccess is a free portal that provides access to the PIDM database. It allows users to retrieve reports on drug safety that have been received by UMC. This definition relies on the System Organ Class (SOC) and Preferred Terms (PTs) of the Medical Dictionary for Regulatory Activities (MedDRA). Therefore, we retrieved records of each anti-VEGF drug and identified the individual AEs using the MedDRA SOC and PT criteria to examine the range of toxicity. The terminology employed in MedDRA is sourced from various dictionaries, including the World Health Organization Adverse Reaction Terms (WHO-ART). According to MedDRA there are 27 SOCs, we analyzed the 27 SOCs that are directly associated with disease symptoms. Our focus was on the PTs, which are publicly accessible aggregated data from the VigiBase database through WHO-VigiAccess.

### Disproportionality analysis

Based on disproportionality analysis, we employed two methods of disproportionate reporting: the reporting odds ratio (ROR) (Rothman et al., 2004), and the proportional reporting ratio (PRR) (Evans et al., 2001). The principles of calculating ROR and PRR are based on the measure of odds imbalance, a method widely used in AE signal mining (van Puijenbroek et al., 2003).

The ROR is calculated to measure the odds imbalance of reporting an AE for a specific drug compared to other drugs. The ROR is given by the formula:

$$ROR=\frac{a\times d}{b\times c}$$

where (*a*) is the number of reports for the specific drug and specific AE, (*b*) is the number of reports for the specific drug and other AEs, (*c*) is the number of reports for other drugs and the specific AE, and (*d*) is the number of reports for other drugs and other AEs. A minimum of 5 cases for the specific drug and AE combination (*a* ≥ 5) is required to ensure the statistical robustness of the ROR calculation.

The PRR is another measure used to quantify the disproportionality of AE reports. It is calculated as:

$$PRR=\frac{a\left(c+d\right)}{c\left(a+b\right)}$$

Similar to ROR, the PRR calculation also requires a minimum of 5 cases for the specific drug and AE combination (*a* ≥ 5) to be considered valid.

A signal is considered disproportionate and potentially indicative of a safety concern if: The ROR value is greater than 2 (ROR > 2), and the lower limit of the 95% Confidence Interval (CI) for ROR is greater than 1 (Lower limit of 95% CI of ROR >1). These criteria ensure that the observed disproportionality is not due to random variability.

The application of ROR and PRR in our analysis allowed us to systematically evaluate the disproportionality of eye disorders reported with the use of anti-VEGF drugs. The results of this analysis contribute to the pharmacovigilance efforts aimed at enhancing drug safety.

### Statistical analysis

In this investigation, a retrospective quantitative research design was utilized. To assess the features of ADR victims for four specific medicines, a thorough descriptive analysis was performed within Excel. The ADR reporting rate of each drug was determined by dividing the total number of ADR symptoms by the total number of ADR reports. The common ADRs for each drug were identified as the symptoms with the highest 20 ADR reporting rates. The occurrence rate of ADR symptoms reported for each drug was calculated, and a comprehensive comparative analysis was carried out. Descriptive variables were classified using frequency and percentage.

## Results

### Overall characteristics of ADR reports for four anti-VEGF drugs

In the WHO-VigiAccess database, the earliest years of ADR reports for ranibizumab, aflibercept, brolucizumab, and faricimab were 2002, 2008, 2018, and 2020, respectively. As of 2023, the WHO has received ADR reports for ranibizumab, aflibercept, brolucizumab, and faricimab were 24,338, 28,524, 4,065, and 852 cases, totaling 57,779 cases. The number of AEs covered in these ADR reports includes 24,305 cases for ranibizumab, 28,454 cases for aflibercept, 4,062 cases for brolucizumab, and 820 cases for faricimab. As shown in Table 2, among the 57,779 reports related to the four anti-VEGF drugs, excluding the 16,856 cases with unknown gender, the number of females experiencing ADRs (22,382 cases, 67.83%) was significantly higher than males (18,586 cases, 32.17%), with a male to female ratio of 2.11:1, indicating a significant gender difference. Excluding reports with unknown age, the age group with the highest incidence rate was over 75 years old. More than half of the AE reports came from the Americas (50.86%), followed by Europe (28.10%). Table 2 also lists the reporting years for each study drug. Ranibizumab and Aflibercept had records before 2010. In the past decade, ranibizumab had a higher number of ADR occurrences in 2014 and 2018; aflibercept had more ADR occurrences in 2018 and 2019 than other years. However, as two newer anti-VEGF drugs for treating wAMD, brolucizumab and faricimab only have data after 2018 and 2020, respectively. The number of ADR occurrences for brolucizumab has been decreasing year by year since 2020; while with the widespread clinical use of faricimab in recent years, the number of ADR occurrences is increasing year by year.

**TABLE 2:** Characteristics of ADR reports of four anti-VEGF drugs.

	Ranibizumab	Aflibercept	Brolucizumab	Faricimab
Number of ADR reports	24,338	28,524	4,065	852
Female	12,293 (50.51%)	7,488 (26.25%)	2,235 (54.98%)	366 (42.96%)
Male	9,656 (39.67%)	7,375 (25.86%)	1,314 (32.32%)	241 (28.29%)
Gender unknown	2,389 (9.82%)	13,661 (47.89%)	516 (12.70%)	245 (28.75%)
<18 years	72 (0.30%)	20 (0.07%)	2 (0.05%)	1 (0.12%)
18–44 years	429 (1.76%)	382 (1.34%)	15 (0.37%)	7 (0.82%)
45–64 years	3,028 (12.44%)	3,078 (10.79%)	324 (7.97%)	59 (6.92%)
65–74 years	3,408 (14.00%)	3,349 (11.74%)	696 (17.12%)	101 (11.85%)
>75 years	8,389 (34.47%)	5,216 (18.29%)	1,171 (28.81%)	228 (26.76%)
Age unknown	9,012 (37.03%)	16,479 (57.77%)	1,857 (45.68%)	456 (53.52%)
Africa	1,059 (4.35%)	1,158 (4.06%)	67 (1.65%)	—
Americas	11,027 (43.31%)	16,238 (56.93%)	1,576 (38.77%)	548 (64.32%)
Asia	3,618 (14.87%)	2,225 (7.80%)	763 (18.77%)	22 (2.58%)
Europe	7,573 (31.12%)	6,864 (24.06%)	1,562 (38.43%)	235 (27.58%)
Oceania	1,061 (6.35%)	2,039 (7.15%)	97 (2.39%)	47 (5.52%)
Before 2010	1,688 (6.94%)	48 (0.17%)	—	—
2011	1,275 (5.24%)	23 (0.08%)	—	—
2012	1,672 (6.87%)	72 (0.25%)	—	—
2013	2,402 (9.87%)	414 (1.45%)	—	—
2014	3,031 (12.45%)	1,495 (5.24%)	—	—
2015	1,568 (6.44%)	1,709 (5.99%)	—	—
2016	1,976 (8.12%)	2,160 (7.57%)	—	—
2017	1,960 (8.05%)	2,820 (9.89%)	—	—
2018	2,941 (12.08%)	5,787 (20.29%)	3 (0.07%)	—
2019	1,459 (5.99%)	5,229 (18.33%)	2 (0.05%)	—
2020	803 (3.30%)	2,373 (8.32%)	1,220 (30.01%)	2 (0.23%)
2021	1,113 (4.57%)	3,116 (10.92%)	1,060 (26.08%)	6 (0.70%)
2022	1,052 (4.32%)	1,705 (5.98%)	1,016 (24.99%)	78 (9.15%)
2023	1,365 (5.61%)	1,503 (5.27%)	761 (18.72%)	734 (86.15%)

### Distribution of SOCs for four anti-VEGF drugs

Table 3 presents the reporting rates of 27 SOCs for four anti-VEGF drugs. Ranibizumab and aflibercept, due to their longer usage duration, have significantly higher incidence rates of diseases in various systems and organs than the other two newer anti-VEGF drugs. The five most common types of AEs are as follows: eye disorders (25,167 cases, 43.56%), general disorders and administration site conditions (19,919 cases, 34.47%), injury poisoning and procedural complications (7,718 cases, 13.36%), infections and infestations (6,710 cases, 11.61%), nervous system disorders (5,774 cases, 9.99%). Given the longer clinical use of ranibizumab and aflibercept, a comparison of these two anti-VEGF drugs reveals that ranibizumab has significantly more cardiac disorders, neoplasms benign malignant and unspecified incl cysts and polyps, whereas aflibercept seems to have more injury poisoning and procedural complications, gastrointestinal disorders, surgical and medical procedures, and social circumstances. Among the reported ADRs in SOC, the incidence rate exceeding 10% is observed in 5 cases of ranibizumab, 4 cases of aflibercept, 2 cases of brolucizumab, and 4 cases of faricimab.

**TABLE 3:** ADR number and report rate of 27 SOCs of four anti-VEGF drugs.

System organ classes	Ranibizumab (N = 24,338)	Aflibercept (N = 28,524)	Brolucizumab (N = 4,065)	Faricimab (N = 852)
Eye disorders	11,043	10,230	3,387	507
General disorders and administration site conditions	8,104	10,815	790	210
Injury poisoning and procedural complications	2,651	4,491	398	178
Infections and infestations	3,141	3,143	309	117
Nervous system disorders	3,226	2,283	200	65
Investigations	1,394	1,584	188	40
Gastrointestinal disorders	847	1,918	54	15
Cardiac disorders	1,738	868	34	24
Vascular disorders	1,073	1,065	127	40
Respiratory thoracic and mediastinal disorders	1,129	941	86	10
Musculoskeletal and connective tissue disorders	778	535	46	12
Neoplasms benign malignant and unspecified incl cysts and polyps	770	368	17	7
Surgical and medical procedures	306	1,000	3	5
Skin and subcutaneous tissue disorders	665	557	70	21
Psychiatric disorders	630	507	86	6
Renal and urinary disorders	528	571	12	9
Metabolism and nutrition disorders	456	444	19	5
Ear and labyrinth disorders	370	231	50	6
Product issues	309	194	14	23
Immune system disorders	239	206	29	7
Blood and lymphatic system disorders	217	541	5	4
Hepatobiliary disorders	103	117	4	1
Reproductive system and breast disorders	61	51	0	2
Social circumstances	54	201	6	2
Congenital familial and genetic disorders	38	22	3	2
Endocrine disorders	34	21	4	0
Pregnancy puerperium and perinatal conditions	34	15	0	0

### Disproportionality analysis based on eye disorders

Eye disorders were found to be the most common AE among the four anti-VEGF drugs by observing and comparing their SOC distribution. To further compare these four drugs, we conducted a disproportionality analysis based on eye disorders. We utilized the methods of ROR and PRR. Table 4 shows that through disproportionality analysis, we found the ROR values of the four drugs to be: ranibizumab: 0.87 (0.85–0.89); aflibercept: 0.62 (0.61–0.64); brolucizumab: 5.54 (5.31–5.79); faricimab: 1.45 (1.32–1.59). The PRR values of the four drugs were: ranibizumab: 0.92 (0.90–0.93); aflibercept: 0.75 (0.74–0.76); brolucizumab: 2.11 (2.08–2.14); faricimab: 1.23 (1.17–1.29). The results indicate that brolucizumab appears to be more likely to cause eye disorders than the other anti-VEGF drugs.

**TABLE 4:** Disproportionality analysis based on eye disorders.

	ROR (95% CI)	PRR (95% CI)
Ranibizumab	0.87 (0.85–0.89)	0.92 (0.90–0.93)
Aflibercept	0.62 (0.61–0.64)	0.75 (0.74–0.76)
Brolucizumab	5.54 (5.31–5.79)	2.11 (2.08–2.14)
Faricimab	1.45 (1.32–1.59)	1.23 (1.17–1.29)

### The most common ocular ADRs of four anti-VEGF drugs

Table 5 lists the 20 most frequently reported ocular ADRs for the four drugs, presented as PTs within the SOC. The common ocular ADRs for ranibizumab include reduced visual acuity (9.81%), visual impairment (8.08%), eye pain (4.63%), blurred vision (4.46%), and eye haemorrhage (3.60%). For aflibercept, the common ocular ADRs are visual impairment (6.19%), blurred vision (4.03%), eye pain (3.80%), cataract (3.12%), and reduced visual acuity (2.95%). Brolucizumab’s common ocular ADRs include vitreous floaters (15.60%), vitritis (15.35%), visual impairment (15.08%), blurred vision (15.01%), and eye inflammation (12.77%). For faricimab, the common ocular ADRs are uveitis (8.10%), visual impairment (7.75%), eye inflammation (7.63%), iridocyclitis (5.63%), and blindness (5.05%).

**TABLE 5:** Top 20 ocular ADRs of anti-VEGF drugs.

Ranibizumab (N = 24,338)		Aflibercept (N = 28,524)		Brolucizumab (N = 4,065)		Faricimab (N = 852)	
ADR	Report rate (%)	ADR	Report rate (%)	ADR	Report rate (%)	ADR	Report rate (%)
Visual acuity reduced	9.81	Visual impairment	6.19	Vitreous floaters	15.60	Uveitis	8.10
Visual impairment	8.08	Vision blurred	4.03	Vitritis	15.35	Visual impairment	7.75
Eye pain	4.63	Eye pain	3.80	Visual impairment	15.08	Eye inflammation	7.63
Vision blurred	4.46	Cataract	3.12	Vision blurred	15.01	Iridocyclitis	5.63
Eye haemorrhage	3.60	Visual acuity reduced	2.95	Eye inflammation	12.77	Blindness	5.05
Cataract	3.12	Eye inflammation	2.91	Uveitis	12.42	Vitritis	5.05
Blindness	3.04	Blindness transient	2.81	Visual acuity reduced	10.77	Vision blurred	4.93
Vitreous floaters	2.45	Blindness	2.66	Retinal vasculitis	9.40	Eye pain	3.99
Ocular hyperaemia	2.21	Blindness unilateral	2.66	Eye pain	9.10	Vitreous floaters	3.29
Retinal haemorrhage	2.06	Vitreous floaters	2.25	Iridocyclitis	7.60	Visual acuity reduced	2.82
Macular oedema	1.73	Eye haemorrhage	1.88	Ocular hyperaemia	6.54	Iritis	2.70
Eye disorder	1.45	Non-infectious endophthalmitis	1.51	Blindness	6.20	Retinal haemorrhage	2.70
Blindness unilateral	1.36	Ocular hyperaemia	1.40	Anterior chamber cell	5.93	Ocular hyperaemia	1.88
Lacrimation increased	1.30	Uveitis	1.26	Keratic precipitates	4.08	Retinal pigment epithelial tear	1.88
Retinal detachment	1.21	Retinal haemorrhage	1.19	Vitreous opacities	3.71	Eye disorder	1.76
Vitreous haemorrhage	1.15	Vitritis	1.12	Retinal artery occlusion	3.52	Cataract	1.53
Eye irritation	1.09	Lacrimation increased	0.96	Retinal vascular occlusion	3.10	Anterior chamber inflammation	1.41
Choroidal neovascularisation	1.08	Eye disorder	0.91	Iritis	2.71	Corneal oedema	1.41
Retinal oedema	1.03	Eye irritation	0.89	Anterior chamber inflammation	2.58	Vitreous opacities	1.41
Subretinal fluid	0.99	Macular oedema	0.72	Retinal haemorrhage	2.53	Photophobia	1.29

Compared to the other three inhibitors, brolucizumab has a significantly higher reported rate of ocular ADRs. Among the most common ocular ADRs of these four anti-VEGF drugs, most are related to visual decline. However, there are some noteworthy events, such as a higher reported rate of cataract when using aflibercept compared to other drugs, and a higher reported rate of uveitis and iridocyclitis when using faricimab compared to other drugs.

### Common and distinct ocular ADRs of four anti-VEGF drugs

By comparing the top 20 ocular ADRs reported for each anti-VEGF drug in SOC, 8 identical ADRs were found among the PTs of the four anti-VEGF drugs (Table 6). These include visual impairment, vision blurred, blindness, vitreous floaters, retinal haemorrhage, visual acuity reduced, eye pain, and ocular hyperaemia. However, there are also some distinct ADR PTs for each of the four anti-VEGF drugs (Table 6). For instance, ranibizumab caused vitreous haemorrhage, retinal oedema, subretinal fluid, choroidal neovascularisation, and retinal detachment; aflibercept caused non-infectious endophthalmitis, and transient blindness; brolucizumab caused retinal vascular occlusion, retinal vasculitis, keratic precipitates, anterior chamber cell, and retinal artery occlusion; faricimab caused corneal oedema, photophobia, and retinal pigment epithelial tear.

**TABLE 6:** The same and different ocular ADRs among four anti-VEGF drugs.

	Ranibizumab	Aflibercept	Brolucizumab	Faricimab
Same ADRs	Visual impairment, Vision blurred, Blindness, Vitreous floaters, Retinal haemorrhage, Visual acuity reduced, Eye pain, Ocular hyperaemia			
Different ADRs	Vitreous haemorrhage, Retinal oedema, Subretinal fluid, Choroidal neovascularisation, Retinal detachment	Non-infectious endophthalmitis, Blindness transient	Retinal vascular occlusion, Retinal vasculitis, Keratic precipitates, Anterior chamber cell, Retinal artery occlusion	Corneal oedema, Photophobia, Retinal pigment epithelial tear

## Discussion

Currently, the monitoring and acquisition of information related to ADRs are primarily through the SRS. The SRS is a system where health professionals or patients voluntarily report ADR information to the ADR database. Despite the rigorous and standardized current drug clinical trials, data from the SRS database can better reflect the safety of a particular drug in the real world compared to clinical trial data. SRS data mainly includes demographic information, drug usage information, ADRs, primary diseases, and reporting sources (Lindquist et al., 2000). VigiBase is the WHO global database for Individual Case Safety Reports (ICSRs), containing ICSRs submitted from medical centers worldwide since 1968 (Tantikul et al., 2008). VigiAccess is a user-friendly web application launched by WHO, allowing public access to VigiBase to provide global drug safety information (Yamoah et al., 2022). This study aims to assess AEs related to four anti-VEGF drugs commonly used to treat wAMD in the VigiAccess database. The results show that the age group with the highest incidence of ADRs related to these four anti-VEGF drugs is over 75 years old. This is mainly because these four anti-VEGF drugs are first-line treatments for wAMD, and the incidence of AMD is mostly over 45 years old and increases with age. Over half of the AE reports came from the Americas (50.86%), followed by Europe (28.10%), possibly due to racial differences in the incidence of AMD, with Caucasians having a significantly higher incidence than people of color. In addition, the number of women experiencing ADRs is significantly higher than men, and the gender ratio difference is large. Many epidemiological surveys on AMD have found that being female is an independent risk factor for AMD disease and progression, which may be due to differences in complement factor levels between men and women (Marin et al., 2022). Since these four drugs are mainly used for the treatment of fundus diseases, the distribution results of the 27 SOCs show that eye diseases are the most common type of AE, accounting for 43.56%, followed by general disorders and administration site conditions (34.47%), and injury poisoning and procedural complications (13.36%). These common AE types are often related to the surgery itself. Due to its unique method of administration through vitreous cavity injection, it requires the operator to have certain experience and skills. Comparing the cases where the incidence of ADRs reported in the SOC exceeded 10%, ranibizumab had 5 cases, aflibercept had 4 cases, brolucizumab had 2 cases, and faricimab had 4 cases. Since ranibizumab and aflibercept have been on the market for more than 10 years and are widely used, while the other two drugs have been on the market for a shorter period of time, we found that ranibizumab had significantly more AEs of cardiac disorders, neoplasms benign malignant and unspecified incl cysts and polyps after comparing it with aflibercept. An Italian study found that the most common AE in 515 patients after ranibizumab injection was cardiac disorders (Menchini et al., 2015), another study based on the FAERS database also showed that cardiac disorders were the most common AE after using ranibizumab (Zhou et al., 2023). The possible reason is that ranibizumab mainly targets VEGF-A, which is involved in protecting cardiomyocytes from damage and promoting their survival (Braile et al., 2020). Therefore, inhibiting this pathway can lead to cardiomyocyte damage and decreased heart function. Our study shows that aflibercept seems to have more gastrointestinal disorders. In a study where aflibercept was used to treat metastatic castration-resistant prostate cancer in men, it was found that the incidence of gastrointestinal disorders was significantly higher in the aflibercept group compared to the placebo group (Tannock et al., 2013). Another clinical study on aflibercept treatment for metastatic colorectal cancer also found that gastrointestinal disorders were the most common AE (accounting for 64.7%) (Ivanova et al., 2017). The gastrointestinal toxicity caused by aflibercept may be related to its inhibition of VEGF, which reduces the blood flow of the digestive tract mucosa, enhances vascular permeability, and causes fluid and protein to leak into the surrounding tissues, thereby causing inflammation and damage to the digestive tract (Cutroneo et al., 2017).

Through the analysis of relevant data from the VigiAccess database, we found that the most common ADRs to ranibizumab were reduced visual acuity, visual impairment, eye pain, blurred vision, and eye haemorrhage. The most common ADRs to aflibercept were visual impairment, blurred vision, eye pain, cataracts, and reduced visual acuity. The most common ADRs to brolucizumab were vitreous floaters, vitritis, visual impairment, blurred vision, and eye inflammation. The most common ADRs to faricimab were uveitis, visual impairment, eye inflammation, iridocyclitis, and blindness. We found that the same ocular ADRs to the four anti-VEGF drugs were often related to decreased vision, eye pain, and vitreous floaters. A recent FAERS study from 2004 Q1 to 2021 Q3 compared the results of ocular AEs associated with anti-VEGF therapy. It showed that the most common ADRs to ranibizumab were macular ischemia and RPE tear, to aflibercept were elevated intraocular pressure and endophthalmitis, and to brolucizumab were retinal vasculitis and/or retinal vascular occlusion, as well as dry eye (Ma et al., 2022). Both WHO-VigiAccess and FAERS can serve as databases for assessing post-marketing drug vigilance. The types and incidence of ocular ADRs caused by anti-VEGF drugs may vary. The FAERS database mainly collects detailed information about drugs on the market in the United States (US) and is more inclined to display specific reports of each ADR, but it requires professionals and intelligent analysis software to screen for eligible case reports.

To further compare the differences between these drugs, we analyzed the differences in ocular ADRs of the four drugs. These ADRs may be related to the molecular weight, structure, mechanism of action, and pharmacokinetics of the drug (Souied et al., 2016). The results showed that the use of ranibizumab led to vitreous haemorrhage, retinal oedema, subretinal fluid, choroidal neovascularisation, retinal detachment, and other ADRs. Due to the downregulation of VEGF’s normal physiological function by anti-VEGF drugs, on the already damaged macular capillary bed, VEGF blockade-induced vasoconstriction may further increase hypoxic injury, causing potential destructive effects on the entire retina including the macular area and the patient’s vision. Compared with the other three anti-VEGF drugs, ranibizumab blocks all subtypes of VEGF and has a Fab fragment, which can more easily penetrate the various layers of the retina. These characteristics may cause corresponding retinal diseases (Ferrara et al., 2006). The use of aflibercept led to non-infectious endophthalmitis, transient blindness, and other ADRs. Research has shown that the main influencing factors of non-infectious endophthalmitis after anti-VEGF injection include patient specificity, drug specificity, and administration specificity (Anderson et al., 2021). Some patients have anti-drug antibodies (ADA) (Wickremasinghe et al., 2008). This ADA titer is related to inflammation and may cause non-infectious endophthalmitis (Baumal et al., 2020). Non-infectious contamination and administration formulas in the drug manufacturing process can also cause non-infectious endophthalmitis (Anderson et al., 2021). The anti-VEGF antibody itself may have immunogenicity. The occurrence of non-infectious endophthalmitis in aflibercept may be related to its special FC antibody part, which interacts with the intra-retinal Fc receptor, triggers an inflammatory response, and may cause non-infectious endophthalmitis (Murinello et al., 2014; Anderson et al., 2021). In addition, protein aggregation or conformational changes may also cause non-infectious endophthalmitis (Melo et al., 2019; Anderson et al., 2021). In February 2020, the American Society of Retina Specialists (ASRS) issued a safety update detailing 14 cases of retinal vasculitis (RV) patients treated with brolucizumab, of which 11 were reported as potentially leading to vision loss due to occlusive retinal vasculitis (ORV). Additionally, Novartis (East Hanover, NJ) received some ADRs about retinal ORV and RV following the market launch of brolucizumab, with a post-marketing incidence of ORV or RO of 10.67/10,000 injections (as of 28 August, 2020). Researchers speculated that the main reason for this AE might be related to the formation of immune complexes caused by ADA in patients (Baumal et al., 2021). A study found that healthy individuals who had not been exposed to brolucizumab already carried brolucizumab-specific B cells in their bodies, and individuals with susceptibility memory to brolucizumab might produce T cell reactivity when re-exposed to this drug. Therefore, immunogenicity may be a prerequisite for the occurrence of RV and RO (Kusuhara et al., 2022). Anette C. Karle’s team and Dominique Brees’ team published two back-to-back papers analyzing potential driving factors for brolucizumab immunogenicity, which might include platelet aggregation, enhanced antigen presentation, cytokine release, and endothelial cell activation (Karle et al., 2023; Kearns et al., 2023). Some previous reports showed that after injection of brolucizumab, intraocular inflammation (IOI) such as keratic precipitates and anterior chamber cell occurred in addition to RV and RO (Kataoka et al., 2021). The possible reason is that brolucizumab is a potent and long-acting anti-VEGF drug, with a higher molar dose in the vitreous cavity and a longer half-life than other anti-VEGF drugs. The prolonged immune response to brolucizumab in the eye and the excessive and persistent VEGF inhibition caused by brolucizumab gradually lead to endothelial cell dysfunction, resulting in thrombosis, vascular leakage, and immune cell migration to the vitreous. This further leads to cumulative damage to the eye tissues. When the damage exceeds a threshold, IOI and/or RV can be clinically detected. Faricimab, as a new generation of intravitreal drugs for the treatment of wAMD, targets not only VEGF but also the ANG-2 receptor, making it a novel treatment approach. Currently, there are not many case reports on its ocular ADRs, and there have been no reports of corneal edema and photophobia, possibly because these ADRs are mild and short-lived. More serious is the occurrence of retinal pigment epithelium (RPE) tear. In 2023, there were reports of cases of RPE tear caused by faricimab (Clemens et al., 2023). Several mechanisms can explain the occurrence of RPE tear after anti-VEGF injection. The most reasonable explanation is that anti-VEGF treatment may lead to fibrotic contraction of the vascularized tissue beneath the RPE, tearing the overlying RPE (Spaide, 2009). Therefore, patients with high-risk factors for RPE tear, including larger pigment epithelial detachment (PED) base diameter, higher PED height, and characteristic changes of RPE such as defects and incisors, should be informed of this risk before treatment (Clemens and Eter, 2016). To minimize the risk of tears, it is also important to extend the injection interval. As faricimab is not widely used clinically at present, and many countries and regions have not yet applied it clinically, further research is needed to confirm its safety, not only its impact on the retina but also its impact on the anterior segment of the eye including the cornea and ocular surface.

Certainly, the use of SRS has certain limitations, such as selection bias in reporting and incomplete information. Although WHO-VigiAccess has been used to mine an increasing number of ADRs, when drugs are launched at different times, the number of ADRs collected varies significantly, making it impossible to compare these drugs simultaneously. Secondly, through VigiAccess, we cannot collect all reports of AEs or medication errors related to a specific drug. Whether an AE or medication error can be reported depends on various factors, such as the time of product launch and the public’s awareness of AEs and medication errors. Thirdly, the causal relationship between AEs and drugs cannot be determined solely based on the data mined. This study collected the number of ADRs and PTs over the years, compared the ADR reporting rates of four anti-VEGF drugs, and tried to avoid the influence of drug launch time. The research results are limited to the relative results of four anti-VEGF drugs, and further clinical research is needed to provide higher-level evidence.

## Conclusion

Anti-VEGF biologics are currently the mainstream treatment for wAMD. This study shows that WHO-VigiAccess has reported over 50,000 ADRs after anti-VEGF antibody treatment, and the most common ADRs are still concentrated in the eye. Compared with the other three inhibitors, Brolucizumab has a significantly higher rate of ocular ADR reports. Although most ocular ADRs are mild and self-limiting, there are also some serious ocular ADRs such as vitreous hemorrhage, RV, RO, endophthalmitis, and even blindness. Countries around the world should actively conduct safety research on biologics, such as cohort event monitoring, to determine the causal relationship between ADRs and drugs. These findings can not only help the public understand the ADRs of anti-VEGF drugs but also assist clinicians in using these expensive drugs more rationally based on the characteristics of each drug’s ADRs, thereby formulating personalized diagnoses and treatment plans for patients.

## Data Availability

The raw data supporting the conclusions of this article will be made available by the authors, without undue reservation.
